# Effect of different salt and fat levels on the physicochemical properties and sensory quality of black pudding

**DOI:** 10.1002/fsn3.390

**Published:** 2016-06-11

**Authors:** Susann Fellendorf, Maurice G. O'Sullivan, Joseph P. Kerry

**Affiliations:** ^1^ The Food Packaging GroupSchool of Food and Nutritional SciencesUniversity College CorkCorkIreland

**Keywords:** Black pudding, traditional food, processed meat, salt and fat reduction

## Abstract

Black pudding, also known as blood sausages or blood pudding, is a kind of meat product made by blood, popular in Asia, Europe, and America. Twenty‐five black pudding formulations with varying fat contents of 2.5%, 5%, 10%, 15%, and 20% (w/w) and sodium contents of 0.2%, 0.4%, 0.6%, 0.8%, and 1.0% (w/w) were manufactured. Sensory acceptance and ranking descriptive analyses as well as compositional and physicochemical analyses were conducted. Samples high in sodium (0.6–1.0%) were scored higher in juiciness, toughness, saltiness, fatness, and spiciness. These samples were the most accepted, whereas samples containing 0.2% sodium were the least accepted. Black pudding samples containing 0.6% sodium and 10% fat displayed a positive (*P* < 0.05) correlation to liking of flavor and overall acceptability. This meets the sodium target level set by the Food Safety Authority of Ireland and shows additionally that a fat reduction in black pudding products is more than achievable.

## Introduction

In the past, food quality was correlated with safety, shelf‐life, and sensory quality. Nowadays food quality, like consumer perception, is difficult to specify as it is permanently dynamic and difficult to measure. Furthermore, it is associated with health and nutrition (Grunert et al. 1996; Peri 2006). Major meat quality cues such as color, visible fat, and drip are important attributes at point of sale, whereas tenderness, flavor, and juiciness play a key role at point of consumption. Additionally, the major background cues for assessors are safety, nutrition, sustainability, and ethics (Steenkamp and Trijp 1996; Acebrón and Dopico 2000; Grunert et al. 2004). All these factors make it necessary for the meat industry to completely understand these cues to satisfy and enhance consumer perceptions. On one hand, meat and meat products are subjected to a negative image which is due in part to issues relating to high saturated fat and salt contents and the association with health issues such as obesity, cardiovascular diseases, and cancer (Li et al. 2005; Cross et al. 2007; Halkjaer et al. 2009; Micha et al. 2010). On the other hand, meat contains high amounts of proteins, essential minerals, and vitamins such as iron, zinc, selenium, vitamins A, B12, and folic acid (Souci et al. 2004; Williams 2007; Biesalski and Nohr 2009). Therefore, muscle‐based food products present something of a conundrum in food product compositional terms. Consumers increasingly reject products containing additives, even when they are deemed to be healthier, though the demand for food high in nutritional value has increased dramatically over the last two decades. Furthermore, the World Health Organization (WHO) have recommended a daily intake of polyunsaturated fatty acids (PUFAs) between 6% and 11% based on daily energy intake (WHO 2003) and have suggested an intake of sodium which is <5 g per day (WHO 2012). A guideline for the Irish meat industry was agreed by the Food Safety Authority of Ireland (FSAI) with the target to decrease the sodium content in black and white puddings to 600 mg/100 g of product (FSAI 2011).

Recipes of black pudding and servings differ dramatically from country to country. In Estonia and Italy, for example, blood sausages are mostly eaten in winter. Black pudding‐style products can contain not only fillings such as breadcrumbs, pine nuts, rice, mashed potatoes, apples, oatmeal, barley, buckwheat, onions, rice, milk, and salt, but also chocolate, raisins, sugar, or butter. Blood sausages are spread on bread, served on bread sticks as a snack, or in slices eaten together with mashed potatoes, fried bacon, beans, eggs, loganberry jam, butter, or sour cream (Predika 1983; Stiebing 1990; Adesiyun and Balbirsingh 1996; Santos et al. 2003; Marianski and Marianski 2011). However, all black pudding‐style products are unified in composition owing to the presence of blood or blood by‐product as an ingredient, thereby providing a unique source of proteins and iron.

In the present study, the focus was directed at black pudding typical of those consumed in Ireland and the United Kingdom, which contains lean pork meat, pork fat, pork blood powder, grains, onions, salt, and seasonings. Usually slices are fried in a pan and served together with bacon, breakfast sausages, eggs, and beans or just with bread. Black puddings have a very high value for the Irish and British as they are particularly a special feature of the traditional Irish and English breakfast. The fat contents of commercially available black puddings range from 7% to 22%, with the majority of products containing between 14% and 16% fat (Fellendorf et al., unpublished data, 2013). The range of sodium contents from commercial product was determined to be between 520 and 1190 mg with an average of 867 mg per 100 g of product (FSAI 2014). Based on the recommendation for salt and polyunsaturated fatty acids levels set by the WHO (WHO 2003, 2012), the sodium guideline for the Irish meat industry set by the FSAI (2011), and the rising demand for healthier products by the consumers, producers are guided to modify their recipes. The stronger interest in health by consumers is already associated with a lower consumption of traditional food products (Pieniak et al. 2009). In this food category, there may exist a conflict between innovation and concept of traditional food (Jordana 2000), which has to be overcome.

Because of health issues relating to higher salt and saturated fat levels in processed meat products and due to the fact that no research has been carried out to date on fat and salt reduction in black pudding products, as determined from extensive review of the scientific literature, the objective of this study was to investigate the interactive effects of varying fat and salt levels on the sensory (sensory acceptance and ranking descriptive analysis [RDA]) and physicochemical properties of black puddings without using additional additives to produce a highly accepted product.

## Materials and Methods

### Sample preparation

Pork trimming lean (visual lean score of 95%) and pork fat were purchased from a local supplier (Ballyburden Meats Ltd., Ballincollig, Cork, Ireland). Meat and fat were minced to a particle size of 10 mm and 5 mm, respectively (TALSABELL SA., Valencia, Spain), vacuum packed, and stored at −20°C. Twelve hours before commencing production, required portions of meat and fat were unfrozen at room temperature until samples reached a working temperature of 4°C. The ingredients were then weighed in accordance with formulations shown in Table 1 for the manufacture of replicated sausage batches. In the bowl chopper (Seydelmann KG, Aalen, Germany) the required meat, fat, seasoning, salt, and three quarters of the required water were added and chopped at high speed (3000 rpm) for 45 sec, followed by adding and mixing the remaining water and blood powder at high speed for 30 sec. The required pinhead oatmeal and dried onions were then chopped at low speed (1500 rpm) for 15 sec and finally, required boiled pearl barley and rusk were chopped at low speed for 30 sec. The black pudding batter was afterward placed into a casing filler (MAINCA, Barcelona, Spain), filled into polyamide casings, and cooked in a Zanussi convection oven (C. Batassi, Conegliano, Italy) using 100% steam at 85°C until the internal product core temperature reached 75°C, as ascertained by a temperature probe (Testo 110, Lenzkirch, Germany). The temperature was held for 15 min and subsequently, the black pudding products were immediately placed in the chill to cool down and stored there at 4°C.

**Table 1:** Black pudding formulations

Sample	Formulation (%)									
	Meat	Fat	Salt	Water	Blood powder	Seasoning	Oatmeal	Onion	Boiled barley	Rusk
F 20 Na 1.0	12.76	36.70	2.54	27.00	3.00	1.10	6.55	2.50	3.00	4.85
F 20 Na 0.8	13.27	36.70	2.03	27.00	3.00	1.10	6.55	2.50	3.00	4.85
F 20 Na 0.6	13.78	36.70	1.52	27.00	3.00	1.10	6.55	2.50	3.00	4.85
F 20 Na 0.4	14.28	36.70	1.02	27.00	3.00	1.10	6.55	2.50	3.00	4.85
F 20 Na 0.2	14.79	36.70	0.51	27.00	3.00	1.10	6.55	2.50	3.00	4.85
F 15 Na 1.0	21.94	27.52	2.54	27.00	3.00	1.10	6.55	2.50	3.00	4.85
F 15 Na 0.8	22.45	27.52	2.03	27.00	3.00	1.10	6.55	2.50	3.00	4.85
F 15 Na 0.6	22.96	27.52	1.52	27.00	3.00	1.10	6.55	2.50	3.00	4.85
F 15 Na 0.4	23.46	27.52	1.02	27.00	3.00	1.10	6.55	2.50	3.00	4.85
F 15 Na 0.2	23.97	27.52	0.51	27.00	3.00	1.10	6.55	2.50	3.00	4.85
F 10 Na 1.0	31.11	18.35	2.54	27.00	3.00	1.10	6.55	2.50	3.00	4.85
F 10 Na 0.8	31.62	18.35	2.03	27.00	3.00	1.10	6.55	2.50	3.00	4.85
F 10 Na 0.6	32.13	18.35	1.52	27.00	3.00	1.10	6.55	2.50	3.00	4.85
F 10 Na 0.4	32.64	18.35	1.02	27.00	3.00	1.10	6.55	2.50	3.00	4.85
F 10 Na 0.2	33.14	18.35	0.51	27.00	3.00	1.10	6.55	2.50	3.00	4.85
F 5 Na 1.0	40.29	9.17	2.54	27.00	3.00	1.10	6.55	2.50	3.00	4.85
F 5 Na 0.8	40.80	9.17	2.03	27.00	3.00	1.10	6.55	2.50	3.00	4.85
F 5 Na 0.6	41.31	9.17	1.52	27.00	3.00	1.10	6.55	2.50	3.00	4.85
F 5 Na 0.4	41.81	9.17	1.02	27.00	3.00	1.10	6.55	2.50	3.00	4.85
F 5 Na 0.2	42.32	9.17	0.51	27.00	3.00	1.10	6.55	2.50	3.00	4.85
F 2.5 Na 1.0	44.87	4.59	2.54	27.00	3.00	1.10	6.55	2.50	3.00	4.85
F 2.5 Na 0.8	45.38	4.59	2.03	27.00	3.00	1.10	6.55	2.50	3.00	4.85
F 2.5 Na 0.6	45.89	4.59	1.52	27.00	3.00	1.10	6.55	2.50	3.00	4.85
F 2.5 Na 0.4	46.40	4.59	1.02	27.00	3.00	1.10	6.55	2.50	3.00	4.85
F 2.5 Na 0.2	46.90	4.59	0.51	27.00	3.00	1.10	6.55	2.50	3.00	4.85

Sample code: F, fat in %; Na, sodium in %.

### Reheating procedure

Before serving black pudding at home, usually the cut slices are cooked in a frying pan. For experimental purpose, the reheating step was standardized with all samples cut into 1.2 cm thick slices, placed on aluminum plates, and dry cooked at 100°C for 7 min in a Zanussi convection oven (C. Batassi) and afterward turned and heated up again at 100°C for an additional 7 min to reach a core temperature of 72°C.

### Sensory evaluation

The sensory acceptance test was conducted using untrained assessors (*n* = 25–28) (Stone and Sidel 2004; Stone et al. 2012a) in the age range of 21–60 years. They were chosen on the basis that they consumed black pudding products regularly. The experiment was conducted in panel booths which conform to the International Standards (ISO 1988). The sensory test followed a balanced block design whereby five reheated samples (coded and presented in a randomised order) were served to the assessors into five sessions. The assessors were asked to assess, on a continuous line scale from 1 to 10 cm, the following attributes: liking of appearance, liking of flavor, liking of texture, liking of color, and overall acceptability (Hedonic). Black pudding samples were presented in duplicate (Stone et al. 2012b). The assessors then participated in a ranking descriptive analysis (RDA) (Richter et al. 2010). They were asked to assess the sensory attributes, such as grain quantity, fatness, spiciness, saltiness, juiciness, toughness, and off‐flavor (intensity), which were also measured on a continuous 10 cm line scale. All samples were again presented randomised in duplicate (session) (Stone et al. 2012b) at ambient temperature.

### Fat and moisture analyses

Moisture and fat content were obtained using the SMART Trac system (CEM GmbH, Kamp‐Lintfort, Germany) (Bostian et al. 1985). Before measuring samples, a reference meat sample with a known fat and moisture content was analyzed to ensure the functionality of the system. Afterward, approximately 1.0 g of the homogenized sample was measured in triplicate, both before and after reheating.

### Protein analysis

Before and after reheating, protein content was also determined using the Kjeldahl method (Suhre et al. 1982). Approximately 1.0–1.5 g of homogenized sample, in triplicate, was weighed into a digestion tube to which two catalyst tabs (each tab containing 3.5 g potassium sulfate and 3.5 mg selenium), 15 mL concentrated sulfuric acid (nitrogen free), and 30% hydrogen peroxide (w/w) were added. In addition, a blank tube was prepared. In the digestion block (FOSS, Tewwtor^™^ digestor, Hillerød, Denmark) the tubes were heated up to 410°C and held for 1 h. After cooling down, 50 mL of distilled water were added to each tube. Before the tubes were placed in the distillation unit (FOSS, Kjeltec^™^ 2100), along with a receiver flask containing 50 mL of 4% boric acid (with bromcresol green and methyl red indicator), 70 mL of 30% sodium hydroxide (w/w) was added before the 5 min distillation started. The content of the receiver flask was then titrated with 0.1 N hydrochloric acid.

### Ash analysis

Before and after reheating, the ash content was determined in triplicate using a muffle furnace (Nabertherm GmbH, Lilienthal, Germany) (AOAC 1923). Approximately 5.0 g of homogenized sample was weighed into porcelain dishes and placed into the muffle furnace. For the preashing step, samples were heated up to 600°C in stages for 12 h and then cooled down. Distilled water was added to the preashed samples in order to increase the surface area of ash particles and heated up again stepwise to 600°C until a white ash was generated.

### Salt analysis

Both, before and after reheating, the salt content of black pudding products were carried out, in triplicate, using the potentiometric method (Fox 1963) which employed a PHM82 Standard pH Meter (Radiometer, Copenhagen) fitted with a combined Ag electrode (M295, Radiometer Analytical SAS, Lyon, France) and a reference electrode Ag/AgCl buffered with KCl (pH C3006, Radiometer Analytical SAS). Approximately 2.0 g of blended samples were weighed into a flask to which 100 mL of 0.1% nitric acid was added. The solutions were then mixed, covered, and placed in a 60°C water bath for 15 min. After cooling down to room temperature, the flasks were potentiometrically titrated with 0.1 N silver nitrate until +255 mV.

### Cooking loss analysis

Before reheating (Reheating procedure), sample weights were recorded. After reheating, the samples were allowed to cool down at room temperature for 20 min and then weighed again to achieve the cooking loss.

### Color analysis

The reheated black pudding samples were analyzed in triplicate using CIE L* a* b* color system by utilizing the Minolta CR 400 Color Meter (Minolta Camera Co., Osaka, Japan) with 11 mm aperture and D_65_ illuminant (International Commission on Illumination, 1976). After calibrating, 10 readings were then conducted per sample.

### Texture analysis

The product texture parameters of hardness, springiness, cohesiveness, and chewiness of the reheated samples were measured in triplicate with texture profile analysis (Bourne 1978) by utilizing the Texture Analyzer 16 TA‐XT2i (Stable Micro Systems, Godalming, UK). Pudding slices were compressed in two cycles using a cross‐head speed of 1.5 mm/sec to 40% of their original size by using a 35‐mm diameter cylindrical probe (SMSP/35Compression plate) attached to a 25‐kg load cell. The following parameters were measured: hardness (N), springiness (dimensionless), cohesiveness (dimensionless), and chewiness (N). The compression of the product in two cycles reflects two human bites with the hardness being the required force to compress food at first bite, which represents the peak force of the first cycle. Springiness describes how well the sample springs back to the original size after deformation, calculated as the ratio of length below graph 2 until maximum force 2 divided by length below graph 1 until maximum force 1. How well the internal structure of a sample withstands compression is expressed by the cohesiveness, which is the ratio of work during compression of the second cycle divided by the first one. Chewiness reflects the required work to chew solid food to a state ready for swallowing, calculated as the product of hardness, springiness, and cohesiveness.

### Data analysis

For evaluating the results of the RDA and the sensory acceptance test, ANOVA partial least square regression (APLSR) was used to process the data accumulated using Unscrambler software version 9.7 (Camo, Norway). The X‐matrix was designed as 0/1 variables for fat and salt contents and the Y‐matrix as sensory variables. Regression coefficients were analyzed by Jack‐knifing, which is based on cross‐validation and stability plots (Martens and Martens 2001). Table 2 displays corresponding *P* values of the regression coefficients. The validated and calibrated explained variances were 16% and 12%, respectively.

**Table 2:** *P* values of regression coefficients from ANOVA partial least square regression (APLSR) for the hedonic and intensity sensory terms of reheated black pudding, including correlation

Sample	Hedonic term					Intensity term						
	Appearance	Flavor	Texture	Colour	Acceptability	Grains	Fatness	Spiciness	Saltiness	Juiciness	Toughness	Off‐flavor
F 20 Na 1.0	0.0014^a^	0.0052^a^	0.0012^a^	0.0022^a^	0.0025^a^	0.0304^b^	0.0081^a^	0.0498^b^	0.0106^b^	0.0021^a^	0.2418 ns	−0.7242 ns
F 20 Na 0.8	0.0151^b^	0.0957 ns	0.0082^a^	0.0145^b^	0.0454^b^	0.3326 ns	0.0007^b^	0.6170 ns	0.1228	0.0011^a^	0.0044^a^	−0.9711 ns
F 20 Na 0.6	0.0542 ns	0.5087 ns	0.0225^b^	0.0492^b^	0.2457 ns	0.8110 ns	0.0008^b^	0.2938 ns	0.5694 ns	0.0008^b^	0.0002^b^	−0.8757 ns
F 20 Na 0.4	−0.4589 ns	−0.3928 ns	−0.2496 ns	−0.4144 ns	−0.7689 ns	−0.1145 ns	−0.0008^b^	−0.0227^b^	−0.2899 ns	−0.0020^a^	−0.0005^b^	0.7165 ns
F 20 Na 0.2	−0.0041ww^a^	−0.0004^b^	−0.0200^b^	−0.0041^a^	−0.0015^a^	−0.0001^b^	−0.0378^b^	−0.0001^b^	−0.0001^b^	−0.0483^b^	−0.0001^b^	0.5253 ns
F 15 Na 1.0	0.0001 b	0.0001^b^	0.0002^b^	0.0001^b^	0.0001^b^	0.0017^a^	0.0047^a^	0.0012^a^	0.0003^b^	0.0011^a^	0.2170 ns	−0.7066 ns
F 15 Na 0.8	0.0003^b^	0.0010^b^	0.0003^b^	0.0006^b^	0.0004^b^	0.0030^a^	0.0089^a^	0.0036^a^	0.0003^b^	0.0015^a^	0.4736 ns	−0.6651 ns
F 15 Na 0.6	0.4595 ns	0.6617 ns	0.3025	0.4186	0.9821	0.2760 ns	0.0093^a^	0.0935 ns	0.5622 ns	0.0116^b^	0.0010^b^	−0.7559 ns
F 15 Na 0.4	0.5053	0.4722 ns	0.2987 ns	0.4687 ns	0.8221 ns	0.1779 ns	0.0021^a^	0.0553 ns	0.3742 ns	−0.0052^a^	0.0016^a^	0.7231 ns
F 15 Na 0.2	−0.0260^b^	−0.0029^a^	−0.0533 ns	−0.0323^b^	−0.0055^a^	−0.0015^a^	−0.1499 ns	−0.0002^b^	−0.0017^a^	−0.5646 ns	−0.0003^b^	0.4886 ns
F 10 Na 1.0	0.0263 b	0.0077^a^	0.0456^b^	0.0304^b^	0.0182^b^	0.0042^a^	0.6154 ns	0.0025^a^	0.0051^a^	0.8051 ns	0.0056^a^	−0.4731 ns
F 10 Na 0.8	0.0029^a^	0.0003^b^	0.0048^a^	0.0038^a^	0.0007^b^	0.0010^a^	0.8392 ns	0.0002^b^	0.0003^b^	0.3407 ns	0.0304^b^	−0.4727 ns
F 10 Na 0.6	0.0264^b^	0.0101^b^	0.0422^b^	0.0358^b^	0.0134^b^	0.0078 a	0.6586 ns	0.0041^a^	0.0034^a^	0.8169 ns	0.0310^b^	−0.4968 ns
F 10 Na 0.4	−0.7403 ns	−0.8510 ns	−0.7157 ns	−0.7320 ns	−0.8109 ns	−0.9219 ns	−0.6268 ns	−0.9756 ns	−0.8649 ns	−0.6292 ns	−0.7184 ns	0.9969 ns
F 10 Na 0.2	−0.0013^a^	−0.0001^b^	−0.0024^a^	−0.0022^a^	−0.0002^b^	−0.0003^b^	−0.7793 ns	−0.0001^b^	−0.0001^b^	−0.2123 ns	−0.0092^a^	0.4753 ns
F 5 Na 1.0	0.0030^a^	0.0004^b^	0.0033^a^	0.0048^a^	0.0006^b^	0.0001^b^	0.9412 ns	0.0001^b^	0.0004^b^	0.3286 ns	0.0166^b^	−0.4901 ns
F 5 Na 0.8	0.9129 ns	0.1708 ns	0.5954 ns	0.8232 ns	0.3506 ns	0.0615 ns	0.0015^a^	0.0170^b^	0.1231 ns	0.0053^a^	0.0022^a^	−0.6523 ns
F 5 Na 0.6	0.8134 ns	0.3378 ns	0.5960 ns	0.7501 ns	0.5785 ns	0.1125 ns	0.0301^b^	0.0385^b^	0.2556 ns	0.0458^b^	0.0063^a^	−0.6912 ns
F 5 Na 0.4	−0.1441 ns	−0.4879 ns	−0.0789 ns	−0.1278 ns	−0.3185 ns	−0.8955 ns	−0.0069^a^	−0.7390 ns	−0.5449 ns	−0.0077^a^	−0.0025^a^	0.9230 ns
F 5 Na 0.2	−0.0003^b^	−0.0005^b^	−0.0001^b^	−0.0018^a^	−0.0005^b^	−0.0014^a^	−0.0009^b^	−0.0004^b^	−0.0001^b^	−0.0001^b^	−0.2470 ns	0.6564 ns
F 2.5 Na 1.0	0.3853 ns	0.0136^b^	0.6522	0.4670 ns	0.0517 ns	0.0062^a^	0.0199^b^	0.0020^a^	0.0205^b^	0.0417^b^	0.0006^b^	−0.5883 ns
F 2.5 Na 0.8	0.7642 ns	0.0769 ns	0.8855 ns	0.8625 ns	0.1926 ns	0.0319^b^	0.0006^b^	0.0037^a^	0.0355^b^	0.0013^a^	0.0001^b^	−0.6302 ns
F 2.5 Na 0.6	0.9538 ns	0.3701 ns	0.7213 ns	0.8870 ns	0.5369 ns	0.1876 ns	0.0602 ns	0.0961 ns	0.2861 ns	0.0755 ns	0.0260^b^	−0.6834 ns
F 2.5 Na 0.4	−0.0063^a^	−0.0873 ns	−0.0018^a^	−0.0072^a^	−0.0269^b^	−0.3848 ns	−0.0001^b^	−0.8200 ns	−0.0904 ns	−0.0001^b^	−0.0007^b^	0.9943 ns
F 2.5 Na 0.2	−0.0002^b^	−0.0001^b^	−0.0001^b^	−0.0002^b^	−0.0001^b^	−0.0005^b^	−0.0074^a^	−0.0001^b^	−0.0001^b^	−0.0002^b^	−0.7429 ns	0.6169 ns

Significance of regression coefficients: ns, not significant.

Sample code: F, fat; Na, sodium.

*P* < 0.05.

a *P* < 0.01.

b *P* < 0.001.

For evaluation the technological data, Tukey's multiple comparison analysis (one‐way ANOVA) was carried out, using Minitab 16 software (Pennsylvania, USA), to separate the averages (*P* < 0.05). The compositional data were evaluated using *t* test, two‐tailed test, and equal variances (software Microsoft [Washington, USA] Excel 2010, *T* test).

## Results and Discussion

### Characterization of black pudding samples

The compositional properties of cooked and reheated black pudding samples with different salt and fat levels are presented in Table 3. The fat levels were slightly higher compared to the designed model (Table 1) as the fat concentration of pure pork fat differs. The marginal higher salt values might be caused by the presence of blood powder, which contains 0.5% salt as a preservative (unpublished data, 2013). In general, reheated black pudding samples had higher (*P* < 0.05) fat, salt, protein, and ash contents, as well as lower (*P* < 0.05) water contents with regard to cooked samples (Table 3). Similar findings were observed in white puddings and pork frankfurters by Fellendorf et al. (2015) and Mittal and Barbut (1994), respectively. Ash contains inorganic substances like sodium chloride wherefore the ash concentration in black pudding increased with increasing the salt content as seen in Table 3.

**Table 3:** Compositional properties of cooked and reheated black pudding samples containing different fat and salt levels

Sample	Fat (%)		Moisture (%)		Protein (%)		Ash (%)		Sodium (%)	
	Cooked	Reheated	Cooked	Reheated	Cooked	Reheated	Cooked	Reheated	Cooked	Reheated
F 20 Na 1.0	23.5 ± 0.1^a^	26.0 ± 0.1^b^	50.7 ± 0.1^a^	46.7 ± 0.1^b^	9.4 ± 0.1^a^	10.0 ± 0.2^b^	3.2 ± 0.0^a^	3.4 ± 0.1^b^	1.06 ± 0.01^a^	1.11 ± 0.01^b^
F 20 Na 0.8	23.0 ± 0.4^a^	26.0 ± 0.2^b^	51.2 ± 0.5^a^	47.3 ± 0.2^b^	9.7 ± 0.0^a^	10.0 ± 0.1^b^	2.6 ± 0.0^a^	2.9 ± 0.0^b^	0.87 ± 0.01^a^	0.91 ± 0.00^b^
F 20 Na 0.6	23.0 ± 0.2^a^	25.8 ± 0.3^b^	51.6 ± 0.2^a^	47.8 ± 0.1^b^	10.2 ± 0.2^a^	10.7 ± 0.2^b^	2.1 ± 0.0^a^	2.3 ± 0.0^b^	0.64 ± 0.01^a^	0.69 ± 0.01^b^
F 20 Na 0.4	23.7 ± 0.2^a^	26.0 ± 0.2^b^	51.2 ± 0.4^a^	47.3 ± 0.2^b^	10.1 ± 0.0^a^	10.8 ± 0.1^b^	1.6 ± 0.0^a^	1.7 ± 0.1^b^	0.45 ± 0.01^a^	0.46 ± 0.00^a^
F 20 Na 0.2	21.9 ± 0.2^a^	24.8 ± 0.2^b^	52.1 ± 0.2^a^	48.0 ± 0.2^b^	10.0 ± 0.2^a^	11.0 ± 0.5^b^	1.1 ± 0.1^a^	1.2 ± 0.0^a^	0.23 ± 0.01^a^	0.26 ± 0.00^b^
F 15 Na 1.0	17.7 ± 0.4^a^	19.7 ± 0.3^b^	54.4 ± 0.3^a^	50.2 ± 0.2^b^	11.1 ± 0.1^a^	11.8 ± 0.5^b^	3.2 ± 0.1^a^	3.4 ± 0.0^b^	1.02 ± 0.01^a^	1.12 ± 0.01^b^
F 15 Na 0.8	17.9 ± 0.1^a^	20.0 ± 0.2^b^	54.2 ± 0.3^a^	50.2 ± 0.1^b^	11.3 ± 0.1^a^	11.9 ± 0.1^b^	2.7 ± 0.0^a^	2.9 ± 0.0^b^	0.83 ± 0.00^a^	0.94 ± 0.01^b^
F 15 Na 0.6	17.7 ± 0.5^a^	19.2 ± 0.1^b^	54.8 ± 0.3^a^	51.3 ± 0.2^b^	11.2 ± 0.1^a^	11.8 ± 0.2^b^	2.2 ± 0.1^a^	2.4 ± 0.0^b^	0.65 ± 0.01^a^	0.74 ± 0.00^b^
F 15 Na 0.4	16.5 ± 0.4^a^	18.3 ± 0.2^b^	56.1 ± 0.4^a^	52.2 ± 0.3^b^	11.5 ± 0.0^a^	12.3 ± 0.0^b^	1.5 ± 0.1^a^	1.8 ± 0.1^b^	0.43 ± 0.00^a^	0.49 ± 0.00^b^
F 15 Na 0.2	17.9 ± 0.2^a^	19.5 ± 0.1^b^	55.8 ± 0.2^a^	51.7 ± 0.1^b^	11.6 ± 0.2^a^	12.6 ± 0.2^b^	1.1 ± 0.0^a^	1.3 ± 0.0^b^	0.22 ± 0.01^a^	0.28 ± 0.01^b^
F 10 Na 1.0	11.7 ± 0.2^a^	13.4 ± 0.1^b^	58.0 ± 0.4^a^	54.0 ± 0.2^b^	12.2 ± 0.1^a^	13.5 ± 0.2^b^	3.2 ± 0.1^a^	3.5 ± 0.1^b^	1.04 ± 0.00^a^	1.16 ± 0.00^b^
F 10 Na 0.8	11.6 ± 0.2^a^	13.1 ± 0.1^b^	58.4 ± 0.3^a^	54.6 ± 0.3^b^	12.4 ± 0.5^a^	13.8 ± 0.1^b^	2.7 ± 0.0^a^	3.0 ± 0.1^b^	0.86 ± 0.01^a^	0.94 ± 0.01^b^
F 10 Na 0.6	12.2 ± 0.1^a^	13.5 ± 0.1^b^	58.5 ± 0.2^a^	54.6 ± 0.1^b^	12.7 ± 0.1^a^	13.8 ± 0.1^b^	2.3 ± 0.1^a^	2.5 ± 0.0^b^	0.64 ± 0.00^a^	0.72 ± 0.01^b^
F 10 Na 0.4	11.9 ± 0.1^a^	13.7 ± 0.2^b^	58.9 ± 0.3^a^	54.8 ± 0.2^b^	12.6 ± 0.1^a^	13.7 ± 0.1^b^	1.7 ± 0.0^a^	1.9 ± 0.1^b^	0.43 ± 0.00^a^	0.51 ± 0.01^b^
F 10 Na 0.2	11.9 ± 0.1^a^	13.6 ± 0.1^b^	58.9 ± 0.3^a^	54.9 ± 0.2^b^	12.7 ± 0.1^a^	14.4 ± 0.2^b^	1.3 ± 0.0	1.4 ± 0.0	0.23 ± 0.00^a^	0.28 ± 0.01^b^
F 5 Na 1.0	6.3 ± 0.1^a^	6.8 ± 0.2^b^	62.4 ± 0.2^a^	58.9 ± 0.3^b^	13.4 ± 0.1^a^	15.0 ± 0.1^b^	3.2 ± 0.1^a^	3.6 ± 0.0^b^	1.01 ± 0.02^a^	1.17 ± 0.01^b^
F 5 Na 0.8	6.7 ± 0.1^a^	7.5 ± 0.1^b^	62.3 ± 0.3^a^	58.7 ± 0.5^b^	13.8 ± 0.1^a^	15.2 ± 0.1^b^	2.9 ± 0.0^a^	3.1 ± 0.0^b^	0.85 ± 0.01^a^	0.93 ± 0.01^b^
F 5 Na 0.6	6.3 ± 0.2^a^	7.0 ± 0.1^b^	62.3 ± 0.3^a^	58.9 ± 0.2^b^	14.0 ± 0.2^a^	15.8 ± 0.1^b^	2.3 ± 0.0^a^	2.5 ± 0.0^b^	0.63 ± 0.00^a^	0.72 ± 0.01^b^
F 5 Na 0.4	6.3 ± 0.1^a^	6.9 ± 0.2^b^	62.4 ± 0.2^a^	59.1 ± 0.1^b^	14.1 ± 0.1^a^	15.8 ± 0.1^b^	1.7 ± 0.1^a^	2.0 ± 0.1^b^	0.42 ± 0.00^a^	0.51 ± 0.01^b^
F 5 Na 0.2	6.7 ± 0.1^a^	7.2 ± 0.1^b^	62.3 ± 0.3^a^	58.9 ± 0.2^b^	14.5 ± 0.0^a^	16.0 ± 0.1^b^	1.3 ± 0.0^a^	1.5 ± 0.0^b^	0.22 ± 0.01^a^	0.27 ± 0.01^b^
F 2.5 Na 1.0	2.4 ± 0.1^a^	2.7 ± 0.3^a^	63.8 ± 0.1^a^	60.1 ± 0.2^b^	14.6 ± 0.1^a^	16.4 ± 0.2^b^	3.3 ± 0.0^a^	3.6 ± 0.1^b^	1.03 ± 0.01^a^	1.14 ± 0.01^b^
F 2.5 Na 0.8	2.1 ± 0.1^a^	2.3 ± 0.2^a^	63.9 ± 0.3^a^	59.9 ± 0.1^b^	14.8 ± 0.1^a^	16.0 ± 0.2^b^	2.8 ± 0.0^a^	3.2 ± 0.0^b^	0.82 ± 0.01^a^	0.90 ± 0.01^b^
F 2.5 Na 0.6	2.7 ± 0.1^a^	3.2 ± 0.2^b^	64.6 ± 0.2^a^	61.1 ± 0.2^b^	14.7 ± 0.1^a^	16.3 ± 0.2^b^	2.4 ± 0.0^a^	2.6 ± 0.0^b^	0.61 ± 0.02^a^	0.72 ± 0.01^b^
F 2.5 Na 0.4	2.7 ± 0.1^a^	3.1 ± 0.1^b^	64.7 ± 0.2	60.9 ± 0.1^b^	14.8 ± 0.1	16.7 ± 0.2^b^	1.9 ± 0.0^a^	2.0 ± 0.0^b^	0.43 ± 0.01^a^	0.48 ± 0.01^b^
F 2.5 Na 0.2	2.8 ± 0.1^a^	3.2 ± 0.1^b^	65.2 ± 0.1^a^	61.5 ± 0.2^b^	15.5 ± 0.1^a^	17.0 ± 0.2^b^	1.3 ± 0.1^a^	1.5 ± 0.0^b^	0.21 ± 0.00^a^	0.31 ± 0.01^b^

All values are averages ± standard errors.

Sample code: F, fat; Na, sodium.

Averages of each cooked and reheated sample of each composition analysis sharing different letters are significantly different (two‐tailed *t* test, *P* < 0.05).

As fat content decreased in black pudding products, a subsequent increase (*P* < 0.05) in cooking loss was observed (Table 4). After reheating, the fat content increased and the water content decreased owing to cooking losses, mainly caused by water losses. Following the recipe for black pudding, less fat was balanced with meat (Table 1). Pure pork fat contains less water than lean pork meats (Souci et al. 2004), consequently, the higher meat content in lower fat black puddings caused higher cooking losses. In previous studies, different findings regarding cooking losses are reported. For instance, Mittal and Barbut (1994) and Fellendorf et al. (2015) found similar results for pork frankfurters and white puddings. In contrast, Ruusunen et al. (2005) recorded increased cooking losses in higher fat ground meat patties. No trend, in terms of cooking loss, was observed in black pudding samples through varying salt contents. This finding is similar to that reported by Fellendorf et al. (2015), but contrasts with those reported with Ruusunen et al. (2005) and Puolanne and Ruusunen (1980). It appears that no general statement can be made about cooking loss and varying salt and fat levels in meat products. Cooking loss might also be influenced by production conditions and processing factors such as chopping time, heating time, cooking temperature, meat type, fat composition, meat product type, ingredient mix, and so on.

**Table 4:** Color, texture profile, and cooking loss values of reheated black pudding samples containing different fat and salt levels

Sample	Color			Texture analyzer				Cooking loss (%)
	L*	a*	b*	Hardness (N)	Springiness	Cohesiveness	Chewiness (N)	
F 20 Na 1.0	24.5 ± 0.3 ^h,i^	4.4 ± 0.1^i,j,k,l^	5.2 ± 0.2^l^	50.3 ± 1.5^j,k^	0.82 ± 0.01^b,c,d^	0.51 ± 0.01^b,c,d,e,f^	21.1 ± 0.9^f,g^	5.7 ± 0.2^g,h,i^
F 20 Na 0.8	25.8 ± 0.3^b,c,d,e,f,g,h^	5.7 ± 0.1^d,e^	6.6 ± 0.2^g,h,i,j^	53.4 ± 1.3^j,k^	0.82 ± 0.01^b,c,d,e^	0.48 ± 0.01^e,f^	21.1 ± 0.8^f,g^	5.4 ± 0.1^h,i^
F 20 Na 0.6	27.7 ± 0.4^a^	6.4 ± 0.1^a,b,c^	7.9 ± 0.2^c,d^	55.6 ± 5.6^i,j^	0.81 ± 0.01^c,d,e^	0.50 ± 0.01^d,e,f^	22.4 ± 0.9^f^	5.1 ± 0.2^i^
F 20 Na 0.4	25.4 ± 0.3^e,f,g,h^	4.7 ± 0.1^h,i,j^	5.7 ± 0.2^k,l^	53.8 ± 1.9^j,k^	0.84 ± 0.01^a,b,c^	0.49 ± 0.01^e,f^	21.9 ± 0.6^f^	5.4 ± 0.2^h,i^
F 20 Na 0.2	25.1 ± 0.4^f,g,h^	4.7 ± 0.1^h,i,j^	6.2 ± 0.3^i,j,k^	35.7 ± 1.0^l^	0.82 ± 0.01^c,d^	0.45 ± 0.01^g,h^	13.1 ± 0.6^h^	5.6 ± 0.2^h,i^
F 15 Na 1.0	27.1 ± 0.4^ab,c^	5.3 ± 0.1^e,f^	7.2 ± 0.2^d,e,f^	79.7 ± 2.0^e,f^	0.82 ± 0.01^b,c,d,e^	0.50 ± 0.00^d,e,f^	32.2 ± 0.9^d,e^	6.3 ± 0.1^g,h,i^
F 15 Na 0.8	26.0 ± 0.3^b,c,d,e,f,g,h^	4.1 ± 0.1^l^	5.9 ± 0.2^j,k,l^	70.0 ± 1.5^g,h^	0.81 ± 0.01^b,c,d,e^	0.49 ± 0.00^e,f^	27.7 ± 0.7^e^	6.7 ± 0.2^g,h^
F 15 Na 0.6	25.4 ± 0.4^e,f,g,h^	4.5 ± 0.1^h,i,j,k^	6.1 ± 0.3^i,j,k^	71.2 ± 1.6^f,g,h^	0.84 ± 0.01^a,b,c^	0.49 ± 0.01^d,e,f^	29.1 ± 1.0^d,e^	6.6 ± 0.2^g,h^
F 15 Na 0.4	26.5 ± 0.4^a,b,c,d,e,f^	4.6 ± 0.1^h,i,j^	6.2 ± 0.3^h,i,j,k^	74.9 ± 4.9^f,g^	0.83 ± 0.01^b,c,d^	0.48 ± 0.01^f,g^	29.6 ± 0.9^g,h^	6.7 ± 0.0^g,h^
F 15 Na 0.2	23.4 ± 0.3^i^	4.8 ± 0.1^g,h,i^	5.5 ± 0.2^k,l^	46.7 ± 1.6^k^	0.80 ± 0.01^c,d,e^	0.44 ± 0.01^h,i^	16.4 ± 0.5^c^	7.0 ± 0.1^f,g^
F 10 Na 1.0	26.7 ± 0.4^a,b,c,d,e^	4.5 ± 0.1^h,i,j,k^	6.6 ± 0.2^f,g,h,i,j^	103.9 ± 2.4^d^	0.85 ± 0.01^a,b,c^	0.51 ± 0.01^b,c,d,e^	45.4 ± 0.4^c^	8.5 ± 0.2^e^
F 10 Na 0.8	27.0 ± 0.4^a,b,c,d^	4.9 ± 0.1^g,h^	7.1 ± 0.2^e,f,g^	109.2 ± 2.0^d^	0.85 ± 0.01^a,b,c^	0.51 ± 0.00^b,c,d,e^	48.0 ± 0.6^c^	8.3 ± 0.2^e,f^
F 10 Na 0.6	24.6 ± 0.3 ^g,h,i^	4.0 ± 0.1^l^	5.8 ± 0.2^j,k,l^	106.0 ± 2.4^d^	0.84 ± 0.01^a,b,c^	0.50 ± 0.01^c,d,e,f^	44.6 ± 1.0^c^	8.7 ± 0.^d,e^
F 10 Na 0.4	25.3 ± 0.3^e,f,g,h^	5.1 ± 0.1^f,g^	7.0 ± 0.2^e,f,g,h^	107.6 ± 1.6^d^	0.84 ± 0.01^a,b,c^	0.51 ± 0.00^b,c,d,e,f^	45.8 ± 1.0^c^	8.7 ± 0.2^d,e^
F 10 Na 0.2	26.3 ± 0.3^a,b,c,d,e,f^	6.6 ± 0.1^a,b^	8.2 ± 0.2^c^	64.2 ± 1.4^h,i^	0.77 ± 0.01^e^	0.42 ± 0.01^i^	20.6 ± 0.6^f,g^	8.4 ± 0.4^e^
F 5 Na 1.0	25.5 ± 0.4^d,e,f,g,h^	4.8 ± 0.1^g,h,i^	7.4 ± 0.3^c,d,e,f^	129.1 ± 2.3^b^	0.85 ± 0.01^a,b,c^	0.51 ± 0.00^b,c,d,e^	56.1 ± 1.3^b^	9.4 ± 0.6^c,d,e^
F 5 Na 0.8	25.4 ± 0.5^d,e,f,g,h^	4.3 ± 0.1^j,k,l^	6.9 ± 0.2^e,f,g,h,i^	129.1 ± 2.3^b^	0.85 ± 0.01^a,b,c^	0.53 ± 0.00^a,b,c^	58.3 ± 1.6^b^	11.1 ± 0.3^a,b^
F 5 Na 0.6	27.2 ± 0.6^a,b^	4.7 ± 0.1^h,i,j^	7.7 ± 0.2^c,d,e^	131.2 ± 2.4^b^	0.85 ± 0.02^a,b,c^	0.52 ± 0.01^a,b,c,d^	57.8 ± 1.9^b^	10.0 ± 0.3^b,c,d^
F 5 Na 0.4	25.1 ± 0.4^f,g,h^	5.8 ± 0.1^d^	8.2 ± 0.2^c^	109.9 ± 1.7^d^	0.84 ± 0.01^a,b,c^	0.49 ± 0.01^d,e,f^	45.6 ± 3.4^c^	10.5 ± 0.2^b,c^
F 5 Na 0.2	25.6 ± 0.5^c,d,e,f,g,h^	6.3 ± 0.2^b,c^	9.0 ± 0.3^b^	83.5 ± 2.5^e^	0.79 ± 0.02^d,e^	0.42 ± 0.01^h,i^	27.8 ± 1.1^e^	10.5 ± 0.2^b,c^
F 2.5 Na 1.0	26.8 ± 0.5^a,b,c,d,e^	4.2 ± 0.1^k,l^	7.4 ± 0.2^c,d,e,f^	146.5 ± 3.4^a^	0.86 ± 0.01^a,b^	0.53 ± 0.01^a,b^	66.9 ± 2.1^a^	10.3 ± 0.3^b,c^
F 2.5 Na 0.8	26.0 ± 0.5^b,c,d,e,f,g,h^	4.3 ± 0.1^j,k,l^	7.2 ± 0.2^d,e,f,g^	119.2 ± 3.0^c^	0.85 ± 0.01^a,b,c^	0.55 ± 0.00^a^	55.6 ± 1.3^b^	10.4 ± 0.2^b,c^
F 2.5 Na 0.6	26.1 ± 0.5^b,c,d,e,f,g^	4.5 ± 0.1^h,i,j,k^	7.5 ± 0.3^c,d,e^	123.8 ± 2.4^b,c^	0.87 ± 0.01^a^	0.55 ± 0.00^a^	59.4 ± 1.1^b^	11.2 ± 0.5^b^
F 2.5 Na 0.4	25.8 ± 0.5^b,c,d,e,f,g,h^	6.0 ± 0.1^c,d^	9.2 ± 0.3^a,b^	102.7 ± 2.0^d^	0.83 ± 0.01^a,b,c,d^	0.51 ± 0.01^b,c,d,e^	44.0 ± 1.4^c^	10.9 ± 0.1^b^
F 2.5 Na 0.2	26.8 ± 0.5^a,b,c,d,e^	6.8 ± 0.1^a^	9.8 ± 0.2^a^	85.9 ± 1.4^e^	0.80 ± 0.02^c,d,e^	0.48 ± 0.01^e,f^	33.5 ± 1.3^d^	12.2 ± 0.1^a^

All values are averages ± standard errors.

Sample code: F, fat; Na, sodium.

Averages sharing different letters in the same column are significantly different (*P* < 0.05).

As shown in Table 4, black pudding samples low in fat (2.5–10%) and sodium (0.2–0.6%) have achieved increases (*P* < 0.05) in yellowness (b) and redness (a) values. Although varying the fat and salt levels has shown no effect on lightness (L). No significant differences in lightness among fat variation were also found by Youssef and Barbut (2011).

### Texture analysis

Table 4 displays the results from the texture profile analysis. Black pudding samples with varied salt and fat contents showed differences (*P* < 0.05) in hardness, springiness, cohesiveness, and chewiness. Lower fat (2.5–10%) samples with 0.2% sodium have shown lower (*P* < 0.05) springiness values. Black pudding samples very low in fat (2.5%) exhibited lower (*P* < 0.05) cohesiveness values, which means less strength of internal bonds compared to samples higher in fat. Samples low in salt (0.2%, 0.4%) were softer (*P* < 0.05) and less chewy (*P* < 0.05). The inverse effect was observed for lower fat samples (2.5–10%). In previous studies, harder and chewier meat products were also reported for samples low in fat (Ahmed et al. 1990; Tobin et al. 2012b, 2013) and high in salt (Tobin et al. 2012a,b; Fellendorf et al. 2015). Samples with varied salt levels achieved consistent instrumental and sensory results for hardness, thus not for samples with varied fat contents (Tables 2, 4). The assessors scored samples high in salt independent of their fat level high for toughness, although the texture analyzer recorded lower (*P* < 0.05) shear force values for higher fat samples. Additional, some of the black pudding samples with similar hardness values were scored differently for toughness. For instance, the sample with 10% fat and 0.8% sodium and the sample with 5% fat and 0.4% sodium achieved hardness values of 109 N, but they were rated high and accordingly low for toughness by the consumers. However, for frankfurters and white puddings inverse results have also been observed (Tobin et al. 2012a; Fellendorf et al. 2015).

### Sensory evaluation

**Figure 1:**
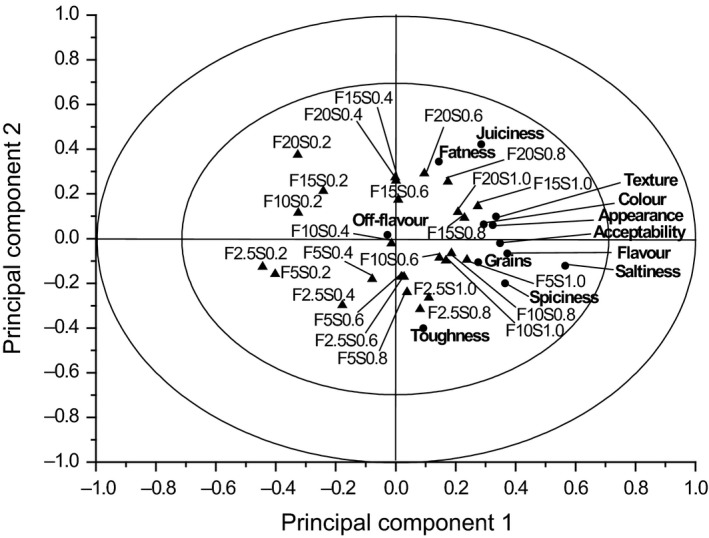
ANOVA partial least square regression (APLSR) plot for the 25 black pudding formulations. ▲ = Samples (F, fat; S, sodium), ● = sensory attributes. Presented is PC1 versus PC 2 for sensory data.

The sensory evaluation of the 25 black pudding products is presented in the APLSR plot in Figure 1 and in conjunction with the ANOVA values of regression coefficients shown in Table 2 for hedonic and intensity sensory terms. The plot gives an overview of the correlation of the attributes and samples. Here, the *x*‐axis of the plot is separated by the *y*‐axis. A positive correlation is presented if the attribute and sample are located on the same side of the *x*‐axis and in close proximity. Furthermore, a negative correlation exists in the inverse case. Table 2 presents the corresponding ANOVA values for Figure 1 and includes significance and correlation factors. A significant difference exists, if the *P* ≤ 0.05. The direction of the correlation (positive or negative) is represented by a+ or − algebraic sign before the *P* value.

As can be seen in Table 2, the assessors liked (*P* < 0.05) the color of black pudding samples containing high sodium (0.6–1.0%) levels. Fat levels, on the other hand, did not affect color acceptance. Instrumental results (Table 4) showed higher (*P* < 0.05) redness (a) and yellowness values (b) for very low fat and salt containing samples, although no trend for lightness (L) values was observed. However, assessors preferred black pudding samples which possessed less yellowness.

From Figure 1, as shown in the right‐hand side quadrant of the figure, liking of appearance was seen to be highly correlated to black pudding products high in fat and salt. Additionally, it is also linked to lower fat (2.5%, 5%) samples containing 1.0% sodium. Samples high in salt and fat (10–20%) were scored higher (*P* < 0.05) in terms of liking of appearance (Table 2). Therefore, liking of product appearance is strongly correlated to the fat and salt content.

Fatness, saltiness, and spiciness are essential attributes for describing flavor. As can be observed in the right‐hand side of Figure 1 and Table 2 the perception of fat, salt, and spices were not affected by fat levels, but were impacted upon by salt levels. The data in Table 2 show that black pudding samples with 0.2% sodium content were negatively (*P* < 0.05) correlated to fatness (with the exception of samples containing 10% and 15% fat), saltiness, and spiciness. Samples of product for all fat levels and with sodium contents of 0.6–1.0% were positively correlated to fatness, saltiness, and spiciness, whereas samples low in sodium (0.2%, 0.4%) were negatively (*P* < 0.05) correlated to these attributes. Wood and Fisher (1990) and Giese (1996) reported that fats interact with other ingredients within a meat system contributing to overall flavor. Moreover, added salt is associated with enhancement of salt perception, flavor perception of further ingredients, and/or bitterness reduction (Hutton 2002; McCaughey 2007). The theory that salt and fat play a key role in enhancing the flavor, can be confirmed only partly by this study. Fellendorf et al. (2015)reported that both salt and fat affect fatness, saltiness, and spiciness perceptions in white pudding. The main differences in black and white pudding composition are the added blood powder, in addition to grains (30% less in black pudding) and the mixture of spices. Blood, and its derivatives, is known for its ability to improve the sensory quality caused by the fat and water‐binding properties of blood proteins and minerals (Pares et al. 2011). In this case, it may be that in addition to fat, ingredients such as salt and spices interact strongly with blood proteins. Seemingly, the lower fat concentration present in black pudding can be compensated by the use of blood powder, therefore both salt and blood powder might act together as a flavor enhancer in black pudding.

From Figure 1, in the right‐hand side quadrant of the figure, liking of texture can be seen to be correlated to black pudding products with higher salt levels. As can be seen in Table 2, samples high in sodium (0.6–1.0%) and fat (10–20%) are positively (*P* < 0.05) correlated to liking of texture, while lower fat and salt samples did not achieve significant positive results, with the exception of the black pudding sample containing 5% fat and 1.0% sodium. Samples of all fat levels, with the exception of 15% fat, with 0.2% sodium content were negatively (*P* < 0.05) correlated to liking of texture. Therefore, varying the salt level in black pudding products affects the sensory acceptance of texture stronger than varying the fat content.

Grains are essential to achieve the typical texture of black pudding, even if the consumer prefers fewer amounts of grains in black pudding compared to white pudding (Fellendorf, et al., unpublished data, 2013). From Figure 1, in the right‐hand side quadrant of the figure, intensity of graininess can be seen to be noncorrelated to the amount of fat in black pudding samples, though it is correlated to salt. Assessors scored samples high in salt positively (*P* < 0.05) to intensity of grains (Table 2). The inverse effect can be observed for samples with the lowest salt concentration (0.2% sodium). These results are not consistent with sample formulations (Table 1) as the amount of added grains is constant. In a previous study by Fellendorf et al. (2015), varying the salt levels in white pudding products was found not to have affected the intensity of graininess. The presence of blood in black puddings may constrict the visual appearance, texture, and taste of the grains used in these products.

As displayed in Figure 1, in the right‐hand side quadrant of the figure, the sensory attributes of juiciness and toughness can be seen to be correlated to samples high in sodium, though no correlation to different fat levels was observed. As seen in Table 2 the majority of the samples high in sodium (0.6–1.0%) for all fat levels were positively (*P* < 0.05) correlated to juiciness and toughness, while samples low in sodium (0.2–0.4%) were rated negatively (*P* < 0.05). In black pudding products, salt influences the attributes of juiciness and toughness. These results are in agreement with Ruusunen et al. (2003) and Matulis et al. (1994) who reported that salt increases juiciness and toughness in frankfurters. Ventanas et al. (2010) showed that the perception of juiciness in cooked bologna‐type sausages was affected by both salt and fat. Furthermore, Fellendorf et al. (2015) observed for white puddings containing fat contents ranging from 5% to 20% and sodium levels ranging from 0.6% to 1.0% that positive correlations to juiciness and negative correlations to toughness were determined. Inverse results were recorded for white pudding samples containing 2.5% fat for all salt levels. However, Hamm (1972) and Desmond (2006) postulated that the relationship between higher salt contents and juiciness, as well as tenderness, is caused by the greater extraction of myofibrillar proteins, resulting in greater water‐binding properties which can be confirmed partly from data generated in the present study.

As can be seen in Table 2, no significant results were achieved for the sensory attribute of off‐flavor. Fellendorf et al. (2015) reported positive (*P* < 0.05) correlations to off‐flavors for lower fat (2.5–5%) white pudding samples containing 0.2% sodium. However, in the present study, varying salt and fat levels in black puddings did not produce any off‐flavors. From Figure 1, in the right‐hand side quadrant of the plot, the attributes for liking of flavor and overall acceptability can be seen to be correlated to not only samples with higher sodium and fat levels, but also to lower fat (2.5–5%) samples containing sodium contents of 0.8% and 1.0%. As shown in Table 2, the concentration of 1.0% sodium in black pudding products for all fat levels (with the exception of the 2.5% fat sample in conjunction with the attribute acceptance) was scored positively (*P* < 0.05) to liking of flavor and overall acceptability. The inverse effect was observed for samples containing 0.2% sodium for all fat levels. Indeed, no correlation was observed between different fat levels and it seems that there exists no observed limit with respect to fat reduction. Salt plays a key role in acceptance and liking of flavor in black puddings. However, the sample with the reduced fat content of 10% and the sodium level of 0.6%, the target level set by the FSAI (2011) achieved positive (*P* < 0.05) correlations to flavor and overall acceptability.

## Conclusion

Fat and salt contents in black puddings have a significant effect on physicochemical and sensory properties, but salt plays the key role. Samples high in sodium (0.6–1.0%) scored higher for juiciness, toughness, saltiness, fatness, and spiciness, and were the most accepted by assessors. Samples with 0.2% sodium were found to be unacceptable. For liking of flavor, no correlation was observed between different fat levels. Thus, there is more opportunity for further potential fat reduction. Black pudding formulations with 0.6% sodium and 10% fat displayed a positive (*P* < 0.05) correlation to liking of flavor and overall acceptability, and thus, meet the sodium target level set by Food Safety Authority of Ireland (FSAI 2011). Additionally, this result shows that a significant fat reduction in black pudding is achievable.

## Conflict of Interest

None declared.
